# The SNP rs402710 in 5p15.33 Is Associated with Lung Cancer Risk: A Replication Study in Chinese Population and a Meta-Analysis

**DOI:** 10.1371/journal.pone.0076252

**Published:** 2013-10-23

**Authors:** Xuzai Lu, Juntao Ke, Xia Luo, Yaowu Zhu, Li Zou, Huijun Li, Beibei Zhu, Zhigang Xiong, Wei Chen, Lingyan Deng, Jiao Lou, Xianxiu Wang, Yu Zhang, Zhenling Wang, Xiaoping Miao, Liming Cheng

**Affiliations:** 1 State Key Laboratory of Environment Health (Incubation), MOE (Ministry of Education) Key Laboratory of Environment & Health, Ministry of Environmental Protection Key Laboratory of Environment and Health (Wuhan), and Department of Epidemiology and Biostatistics, School of Public Health, Tongji Medical College, Huazhong University of Science and Technology, Wuhan, China; 2 Department of Laboratory Medicine, Tongji Hospital, Tongji Medical College, Huazhong University of Science and Technology, Wuhan, China; 3 Guangdong Maternal and Child Health Care Hospital, Guangzhou, China; Faculty of Medicine, University of Porto, Portugal

## Abstract

**Background:**

Lung cancer is the most commonly diagnosed cancer and leading cause of cancer mortality in the world. A single nucleotide polymorphism (SNP), rs402710, located in 5p15.33, was firstly identified to be associated with the lung cancer risk in a genome-wide association study. However, some following replication studies yielded inconsistent results.

**Methodology and Findings:**

A case-control study of 611 cases and 1062 controls in a Chinese population was conducted, and then a meta-analysis integrating the current and previously published studies with a total 31811 cases and 36333 controls was performed to explore the real effect of rs402710 on lung cancer susceptibility. Significant associations between the SNP rs402710 and lung cancer risk were observed in both case-control study and meta-analysis, with ORs equal to 0.77 (95%CI = 0.63–0.95) and 0.83 (95%CI = 0.81–0.86) in dominant model, respectively. By stratified analysis of our case-control study, the associations were also observed in never smoker group and non-small cell lung cancer(NSCLC) group with ORs equal to 0.71 (95%CI = 0.53–0.95) and 0.69 (95%CI = 0.55–0.87), which was remarkable that larger effect of the minor allele T was seen in the two groups than that in overall lung cancer. Besides, the sensitive and cumulative analysis indicated the robust stability of the current results of meta-analysis.

**Conclusion:**

The results from our replication study and the meta-analysis provided firm evidence that rs402710 T allele significantly contributed to decreased lung cancer risk, and the case-control study implied that the variant may yield stronger effect on NSCLC and never smokers. However, the mechanism underlying the polymorphism conferring susceptibility to lung cancer is warranted to clarify in the follow-up studies.

## Introduction

Lung cancer is the most commonly diagnosed cancer and leading cause of cancer mortality worldwide, with 1.61 million newly confirmed cases and 1.38 million death from lung cancer annually [1].There are multiple factors attributing to lung cancer, of which environmental exposure, primarily to smoking, is the major risk factor. However, not all lung cancers are due to smoking, and increasing evidence for the association between genetic factors and lung cancer risk is being identified by hundreds of studies [2], [3]. The existing evidence suggested that the genetic factors may play a very important role in the development of lung cancer.

Genome-wide association studies (GWAS), which aim to cover most genetic variation by genotyping up to 1,000,000 single nucleotide polymorphisms (SNPs) and do not require prior knowledge of gene function, are efficiently applied to identify the association between common genetic variants and complex disease [4], [5], [6], [7]. Recently multiple genome-wide association studies have identified multiple novel SNPs on chromosome 15q25 [8], [9], [10], 5p15 [11], [12] and 6q21 [11], associated with lung cancer. Among these SNPs, rs402710 on 5p15.33, located in the intron region of cleft lip and palate transmembrane 1-like (*CLPTM1L*), was firstly identified in a GWAS set of 3,259 cases and 4,159 controls and a replication set of 2,899 cases and 5,573 controls by McKay JD et al [12]. *CLPTM1L*, alias *CRR9*, which was found to express in various tissue, including lung tissue and overexpress in cisplatin-resistant cell lines, encodes an enzyme—cleft lip and palate trans-membrane 1-like that may be associated with apoptosis [13]. In consideration of the premises, this associated SNP rs402710 has attracted many investigators' attention from multiple countries and regions. Several follow-up replication studies have resoundingly replicated the significant association of the SNP with lung cancer risk, in Caucasian [14], [15], [16] and Asian [16], [17], [18], [19] population. However, some other replication studies showed the inconsistent outcomes [20], [21], [22]. Two Chinese replication studies failed to identify the similar effect in separate Chinese population [20], [21], which may be due to the small sample size. Additionally, owing to the phenomena “winner's curse” that the effect sizes of initial positive study are usually overestimated, the following replication studies are possibly to be underpowered and then very likely to fail if the necessary sample sizes are based on the initially overestimated effect sizes [23]. Nevertheless, meta-analysis, a method combining data together to make sample size exponential growth to get enough power, can clarify inconsistent results in genetic association studies [24]. Therefore, we conducted a case-control study to examine the association between rs402710 and lung cancer risk in Chinese population, after that, a meta-analysis combining previously published studies and our current study was conducted to provide a more precise estimate of this association.

## Materials and Methods

### Study population

Between 2009 and 2011, a total of 611 newly confirmed cases and 1062 cancer-free controls were obtained from the Tongji Hospital of Huazhong University of Science and Technology (HUST). All of them were genetically unrelated ethnic Han Chinese residing in Wuhan City or surrounding regions in Hubei Province of central China. The 611 cases were histopathologically confirmed without any treatment (such as radiotherapy or chemotherapy) prior to blood samples collection. All controls were randomly selected from the individuals who participated in a health check-up program at the same hospital during the same period as the cases were recruited. The controls had no history of cancer and were frequency matched to the cases by age (±5 years), sex, gender and residential area (urban or rural). After a written informed consent was obtained, a 5-ml peripheral blood sample and a questionnaire were collected from each subject. Smoker was defined as one person who had smoked at least one cigarette per day>1 year or longer at any time in their life, while never-smokers were defined as those who had not. This study was approved by ethics committee of Tongji Hospital of Huazhong University of Science and Technology.

### Genotyping

Genomic DNA was extracted from 5-mL of peripheral blood sample using the Relax Gene Blood DNA System DP319-02 (Tiangen, Beijing, China) according to the manufacturer's instructions. The genotypes of rs402710 SNP was performed by the TaqMan SNP Genotyping Assay(Applied Biosystems, Foster city, CA) using the 7900HT Fast Real-Time PCR System (Applied Biosystems, Foster city, CA) without knowing the subjects' case or control status. To validate the results, 5% duplicated samples were randomly selected to assess the reproducibility, with a concordance rate of 100%.

### Statistical analysis

Differences in the distributions of demographic characteristics, selected variables and genotypes between cases and controls were examined by *χ^2^* test and *t* test. The Hardy–Weinberg equilibrium (HWE) was tested by a goodness of fit *χ^2^* test in the control subjects. Unconditional logistic regression was used to estimate crude odds ratio (OR), adjusted OR and their 95% confidence intervals (CIs) for the effect of rs402710 genotypes on lung cancer risk, with adjustment for age, sex and smoking status, where appropriate. In order to avoid the assumption of genetic models, dominant (TT plus CT vs CC), recessive (TT vs CT plus CC) and additive models were also analyzed. All the analyses were performed using SPSS version 19.0. The criterion of statistical significance except for heterogeneity test was *P*<0.05, and all statistical tests were two sided.

### Meta-analysis of rs402710 in association with lung cancer risk

Followed the methods proposed by the Preferred Reporting Items for Systematic Reviews and Meta-Analyses (PRISMA) [25] and Meta-analysis Of Observational Studies in Epidemiology (MOOSE) guidelines [26], we using the search terms *rs402710, 5q15.33* or *CLPTM1L*, combined with *lung cancer, lung neoplasia, lung adenoma*, *lung carcinoma*, or *lung tumor* in the PubMed, Embase and ISI Web of Knowledge databases for published studies without language restriction. References cited by the retrieved articles were also scanned for additional information. Eligible studies had to meet the following criteria: (a) a case-control or nested case-control study assessing the association between rs402710 and lung cancer risk; (b) contained sufficient information about genotype or allele frequency for risk estimates, or original data through which we can calculate what we need; (c) genotypes in controls were in Hardy-Weinberg equilibrium (*P*>0.01); (d) studies of humans. If there was parallel publication, we selected the study with larger sample size. If more than one ethnic population were involved in one report, each population was considered separately.

The following data were extracted from eligible study by two independent authors (Xuzai Lu & Xia Luo): first author, year of publication, geographic location, ethnicity of study population, study design, genotyping method, numbers of cases and controls, male/female rate, mean age, family history of cancer, source of control group, frequencies of genotypes in cases and controls. ORs and 95% CIs as the metrics of effect size were re-calculated for the allele T versus C, genotypes CT versus CC and TT versus CC and so were the dominant, recessive and additive models respectively. The *χ^2^*-based Cochran's *Q* statistic test was utilized to test heterogeneity (true variance of effect size across studies), and we considered that the heterogeneity was significant if *P*<0.100 for *Q* statistic [27]. Subsequently, the *I^2^* statistic, which reveals the proportion of the variability in effect estimation, was employed to quantify heterogeneity among studies (*I^2^* = 0–25%, no heterogeneity; *I^2^* = 25–50%, moderate heterogeneity; *I^2^* = 50–75%, large heterogeneity; *I^2^* = 75–100%, extreme heterogeneity) [28]. When the heterogeneity was negligible (*P*>0.1 for *Q* statistic), we pooled the data from studies by a fix-effects model using Mantel-Haenszel method, otherwise, we employed a random-effects model using DerSimonian and Laird method [29]. To explain sources of heterogeneity cross studies, stratified analysis, according to ethnicity (Asian and Caucasian), sample size (≤1000 and >1000), was performed. Subsequently, cumulative meta-analysis was conducted to observe the trend of association between rs402710 and lung cancer risk in chronological order of the eligible literatures [30]. Sensitivity analysis was employed to estimate respective influence of each study on overall estimate by omitted each study in turn [31]. Publication bias was detected by funnel plot [32], whose asymmetry was investigated with Egger's test and Begg's test [33] and a trim and fill method [34], if necessary (*P*<0.05 for Egger's test or Begg's test), was applied. All statistical analyses were carried out by Stata Version 11.0. All *P* values are two-tailed with a significant level at 0.05. If we got a significant association between the SNP and lung cancer risk, bioinformatics analyses were further carried out to predict the function of rs402710 using three comprehensive bioinformatics tools “FastSNP” (http://fastsnp.ibms.sinica.edu.tw/pages/input_CandidateGeneSearch.jsp), “SNP Info” (http://manticore.niehs.nih.gov/snpfunc.htm) and “F-SNP” (http://compbio.cs.queensu.ca/F-SNP/).

## Results

### Results of case-control study

#### Characteristics of study population

A total of 611 lung cancer cases and 1062 cancer-free controls were enrolled in our study. The characteristics of all subjects were listed in Table 1. Males were 68.6% among cases compared with 70.2% among controls (*P* = 0.475). The mean age was 60.97 (±10.76) years for cases and 61.71 (±9.36) years for controls (*P* = 0.145). There was also no significant difference in distribution of age (<50 and ≥50 years old, *P* = 0.070). More smokers were observed in cases compared with subjects in control group (*P*<0.001), considering that most lung cancers are attributable to smoking. 421 (68.9%) of the cases were histopathologically confirmed as non-small-cell lung cancer (NSCLC), including squamous cell carcinomas, adenocarcinomas and large cell lung carcinomas, meanwhile NSCLC accounts for approximately 80% of primary lung cancers in general.

**Table 1:** The characteristics of our study population.

Variables	Case (N = 611) No. (%)	Control (n = 1062) No. (%)	*P*
gender			0.475^a^
Male	419(68.6)	746(70.2)	
Female	192(31.4)	316(29.8)	
Median age	60.97±10.76	61.71±9.36	0.145^b^
<50.0	85(13.9)	116(10.9)	0.070^a^
≥50.0	526(86.1)	946(89.1)	
Smoking status			<0.001^a^
Never-smoker	279(45.7)	599(56.4)	
Smoker	324(53.0)	458(43.1)	
Unknown	8(1.3)	5(0.5)	
Subtype			
NSCLC	421(68.9)		
Others	190(31.1)		

a *P* value was calculated by the *x* ^2^ test;

b *P* value was calculated by the *t* test.

#### Association analysis

The call rate of genotyping was 99.6% for the SNP rs402710. The distribution of the SNP genotypes in cases and controls was showed in Table 2. Genotypes in controls were in accordance with Hardy-Weinberg equilibrium (*P* = 0.955). Significant difference was observed in distribution of genotypes between cases and controls (*χ^2^* = 4.368, *P* = 0.037).

**Table 2:** Association between rs402710 and lung cancer risk in a Chinese population^a^.

	Case	Control	OR(95%CI)					
	CC/CT/TT	CC/CT/TT	T vs C	CT vs CC	TT vs CC	Dominant model	Recessive model	Additive model
Total^b^	321/224/59	494/461/107	0.85(0.73–0.99)	0.75(0.61–0.93)	0.85(0.60–1.20)	0.77(0.63–0.94)	0.97(0.69–1.35)	0.85(0.73–0.99)
Total	321/224/59	494/461/107	0.86(0.73–1.00)	**0.75(0.60–0.93)**	0.87(0.61–1.24)	**0.77(0.63–0.95)**	0.99(0.71–1.39)	**0.85(0.73–0.99)**
Smoking status								
Smoker	164/126/31	215/194/49	0.88(0.71–1.10)	0.85(0.63–1.15)	0.82(0.50–1.34)	0.84(0.63–1.12)	0.88(0.55–1.42)	0.89(0.72–1.10)
Never smoker	153/94/28	277/264/58	0.83(0.66–1.04)	**0.66(0.48–0.90)**	0.93(0.56–1.53)	**0.71(0.53–0.95)**	1.11(0.68–1.80)	0.81(0.65–1.01)
NSCLC								
total	236/146/39	494/461/107	**0.79(0.66–0.94)**	**0.67(0.53–0.86)**	0.78(0.52–1.17)	**0.69(0.55–0.87)**	0.93(0.63–1.37)	**0.78(0.66–0.93)**
Smoking status								
Smoker	123/85/23	215/194/49	0.84(0.66–1.08)	0.76(0.55–1.07)	0.80(0.47–1.39)	0.77(0.56–1.06)	0.91(0.54–1.53)	0.85(0.67–1.08)
Never smoker	112/59/16	277/264/58	**0.72(0.55–0.94)**	**0.57(0.40–0.82)**	0.74(0.40–1.35)	**0.60(0.43–0.84)**	0.93(0.52–1.68)	**0.70(0.53–0.91)**

Abbreviations: OR, Odds ratio; 95%CI, 95% confidence interval.

a ORs and their corresponding 95% CIs were calculated by multivariate logistic regression model after adjusting for age, sex and smoking.

b Crude ORs and their corresponding 95% CIs were calculated without adjustment.

By multivariate logistic regression model adjusted for age, sex and smoking status, the significant association between rs402710 and lung cancer was observed in heterozygote model (CT versus CC: OR = 0.75, 95%CI = 0.60–0.93), which indicated that the individuals with the CT genotype had a significantly decreased risk of lung cancer compared to the CC genotype carriers. Likewise, significant associations were found in dominant and additive models (dominant model: OR = 0.77, 95%CI = 0.63–0.95, additive model: OR = 0.85, 95%CI = 0.73–0.99). A dominant model was a T carrier (TT plus CT) group which was combined the TT carriers with the CT carriers to increase statistical power. The per-T-allele OR could be calculated in an additive model. Considering smoking is a major factor contributing to lung cancer, we respectively stratified the cases and controls into two groups, smoker and never smoker, to detect the association between rs402710 and lung cancer. The same effect between rs402710 and lung cancer was also observed in heterozygote and dominant models in never smoker group (heterozygote model: OR = 0.66, 95%CI = 0.48–0.90; dominant model: OR = 0.71, 95%CI = 0.53–0.95). However, no valid associations were found under any models of smoker group, suggesting that the T carriers who never smoke might have less risk of lung cancer than those smokers.

To explore the rs402710's effect on NSCLC, we also compared the genotype distribution between the NSCLC cases and controls. Like total lung cancers, similar positive association and stronger genetic effect were found in three models of NSCLC (heterozygote model: OR = 0.67, 95%CI = 0.53–0.86; dominant model: OR = 0.69, 95%CI = 0.55–0.87; additive model: OR = 0.78, 95%CI = 0.66–0.93). It is remarkable that larger effect of the minor allele T was seen in NSCLC than that in overall lung cancer. But beyond that, in the allelic model, T allele carriers also showed significantly protective effect compared to those with the C allele (OR = 0.79, 95%CI = 0.66–0.94). Subsequently, the NSCLC and controls were once again stratified into two groups: smoker and never smoker Likewise, under the same four models of never smoker group, positive association and stronger genetic effect were found. However, significant associations were still not detected under any models of smoker group.

### Results of meta-analysis

#### Characteristic of included studies

As show in Figure S1, we found 16 potentially relevant reports after comprehensive searching, of which, 2 reports with insufficient information were omitted after contacting with authors by e-mail [35], [36]. However, the data of a report accomplished by Xun et al [37] completely came from a GWA study by McKay et al [12], which firstly identified the association between rs402710 and lung cancer risk. One study conducted by Truong et al [16] contained two ethnicities of Caucasian and Asian. Finally, as shown in Table 3, 13 reports plus our case-control study comprising 21 studies of 31811 cases and 36333 controls were included in this meta-analysis [12], [14], [15], [16], [17], [18], [19], [20], [21], [22], [38], [39], [40], involving 13 Caucasian studies and 8 Asian studies.

**Table 3:** Characteristics of studies on rs402710 polymorphisms and risk of lung cancer included in the meta-analysis.

First author	Year	Country	Ethnicity	Study method	Study design	Genotyping	Tumor subtype	Case/control
James D McKay	2008	France	Caucasian	Cohort/CC	GWAS+Replication	Illumina/Taqman	Lung cancer	5223/7624
Guangfu Jin	2009	China	Asian	CC	Replication	PCR	NSCLC	1199/1329
Shanbeh Zienolddiny	2009	Norway	Caucasian	CC	Replication	Taqman-PCR	NSCLC	356/432
Kyon-Ah Yoon	2010	Korea	Asian	CC	GWAS/Replication	Affymetrix/Taqman	NSCLC	1425/3011
Young	2010	New Zealand	Caucasian	CC	Replication	Sequenom	Lung cancer	453/487
Chao Agnes Hsiung	2010	TaiWan	Asian	Cohort/CC	GWAS+Replication	Illumina/Taqman	AD	2659/2844
Therese Truong(ERU)	2010	USA	Caucasian	CC	Replication	Mix	Lung cancer	8860/9198
Therese Truong(Asian)	2010	USA	Asian	CC	Replication	Mix	Lung cancer	1680/2117
Ping Yang(A)	2010	Ireland	Caucasian	CC	Replication	Taqman-PCR	Lung cancer	1735/1036
Ping Yang(B)	2010	Ireland	Caucasian	CC	Replication	Taqman-PCR	Lung cancer	651/1206
Ping Yang(C)	2010	Ireland	Caucasian	CC	Replication	Taqman-PCR	Lung cancer	1406/412
Ping Yang(D)	2010	Ireland	Caucasian	CC	Replication	Taqman-PCR	Lung cancer	415/614
Ping Yang(E)	2010	Ireland	Caucasian	CC	Replication	Taqman-PCR	Lung cancer	771/260
Ping Yang(F)	2010	Ireland	Caucasian	CC	Replication	Taqman-PCR	Lung cancer	329/624
Ping Yang(G)	2010	Ireland	Caucasian	CC	Replication	Taqman-PCR	Lung cancer	82/133
Pande	2011	USA	Caucasian	CC	Replication	Illumina	Lung cancer	1074/1091
XF Chen	2011	China	Asian	CC	Replication	Taqman-PCR	AD	225/193
Ewa Jaworowska	2011	Poland	Caucasian	CC	Replication	Taqman-PCR	Lung cancer	848/845
Eun Young Bae	2012	Korea	Asian	CC	Replication	PCR	Lung cancer	1094/1099
Hidemi Ito	2012	Japan	Asian	CC	Replication	Taqman-PCR	Lung cancer	716/716
Lu	2012	China	Asian	CC	Replication	Taqman-PCR	Lung cancer	611/1062

NSCLC: non-small-cell lung cancer; AD: adenocarcinomas.

#### Pooled frequency of risk allele

HapMap database displayed that the T allele frequencies of rs402710 in Caucasians, Chinese and Japanese were 36.5%, 31.5% and 30.9% respectively. And we similarly found the pooled T allele frequencies were 35.0% (95%CI = 34.5%–35.4%) in Caucasian controls and 31.7% (95%CI = 30.6%–32.9%) in Asian controls in our meta-analysis, under fixed and random model respectively.

#### Overall meta-analysis of rs402710 in associated with lung cancer

**Figure 1:**
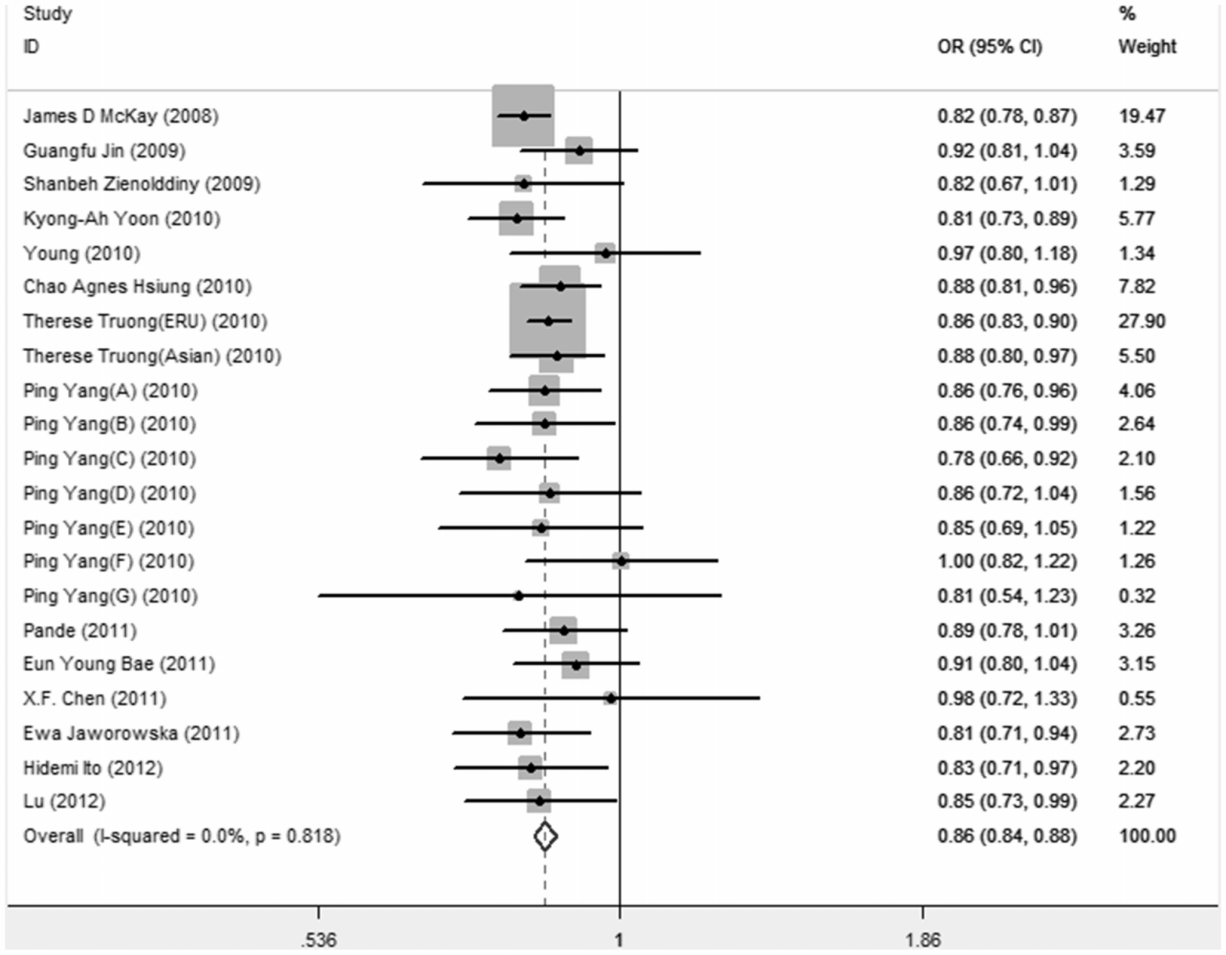
Forest plot of association of rs402710 with lung cancer under allelic model.

As shown in Table 4, no significant evidence of heterogeneity was detected in all genetic models (*P*>0.10), therefore a fix-effects model was employed to pool the OR for all models. In allelic model, as shown in figure 1, the T allele presented a pooled OR of 0.86 (95%CI = 0.84–0.88) compared to the C allele. Genotypic ORs of the TT versus CC and CT versus CC were 0.73(95%CI = 0.69–0.77) and 0.86(95%CI = 0.83–0.89). Similarly, the dominant, recessive and additive models were all significantly associated with lung cancer risk (dominant model: OR = 0.83, 95%CI = 0.81–0.86; recessive model: OR = 0.79, 95%CI = 0.75–0.83; additive model: OR = 0.86, 95%CI = 0.84–0.88).

**Table 4:** Pooled OR with 95% CI for the association between rs402710 and lung cancer risk in the meta-analysis.

	Genetic model	OR(95%CI)	*P*	*I^2^*	*P* for Heterogeneity	*P* for Egger's test	*P* for Begg's test
Overall							
N = 21	T vs C	0.86(0.84–0.88)	<0.001	0%	0.818	0.296	0.833
	TT vs CC	0.73(0.69–0.77)	<0.001	0%	0.602	0.777	0.880
	CT vs CC	0.86(0.83–0.89)	<0.001	0%	0.566	0.335	0.740
	Dominant	0.83(0.81–0.86)	<0.001	0%	0.734	0.276	0.740
	Recessive	0.79(0.75–0.83)	<0.001	1.4%	0.440	0.977	0.833
	Additive	0.86(0.84–0.88)	<0.001	0%	0.810	0.444	1.000

#### Stratified analyses

We performed stratified analyses to explore the potential source of heterogeneity by ethnicity and sample size (Table 5). After stratified by ethnicity, no significant between-study heterogeneity was detected in Caucasian (*P _heterogeneity_*>0.1, all *I^2^* = 0%), meanwhile the heterogeneity in heterozygous (*I^2^* = 38.7%) and recessive (*I^2^* = 39.3%) model among Asian studies slightly increased from no heterogeneity grade to moderate grade. It might be due to that the heterogeneity largely came from Asian studies and the heterogeneity in Caucasian studies was little. Even the heterogeneity in Asian studies which could be accounted for different study design and genotyping methods reached moderate grade, the values of *P* for heterogeneity were all greater than 0.10. In other words, the results of meta-analysis in Asian studies were credible and representative. In spite of this, the significant association between the SNP rs402710 and lung cancer risk was still identified in all genetic models of both Caucasian and Asian groups. Subsequently, the data of genotypes was stratified by sample size, which was defined large group when it was more than 1000, otherwise was small group. In the two subgroups, all genetic models exhibited significant association with decreased lung cancer risk, and showed no significant between-study heterogeneity.

**Table 5:** Stratified analysis of the association between rs402710 genotype and lung cancer risk.

	Genetic model	OR(95%CI)	*P*	*I^2^*	*P* for Heterogeneity	*P* for Egger's test	*P* for Begg's test
Asian							
N = 8	T vs C	0.87(0.84–0.91)	<0.001	0%	0.695	0.592	1.000
	TT vs CC	0.75 (0.68–0.82)	<0.001	15.0%	0.312	0.710	0.902
	CT vs CC	0.89(0.84–0.94)	<0.001	38.7%	0.121	0.526	1.000
	Dominant	0.86(0.81–0.91)	<0.001	5.0%	0.391	0.498	1.000
	Recessive	0.79(0.72–0.87)	<0.001	39.3%	0.117	0.650	0.902
	Additive	0.87(0.84–0.91)	<0.001	0%	0.691	0.650	1.000
Caucasian							
N = 13	T vs C	0.85(0.83–0.88)	<0.001	0%	0.733	0.549	0.951
	TT vs CC	0.73(0.68–0.77)	<0.001	0%	0.675	0.703	1.000
	CT vs CC	0.85(0.81–0.88)	<0.001	0%	0.953	0.624	0.855
	Dominant	0.82(0.79–0.85)	<0.001	0%	0.889	0.578	0.855
	Recessive	0.79(0.74–0.84)	<0.001	0%	0.732	0.738	0.760
	Additive	0.85(0.83–0.87)	<0.001	0%	0.745	0.754	0.951
Large size							
N = 9	T vs C	0.86(0.84–0.88)	<0.001	5.3%	0.495	0.237	0.175
	TT vs CC	0.73(0.69–0.78)	<0.001	5.3%	0.489	0.276	0.048
	CT vs CC	0.86(0.83–0.89)	<0.001	0%	0.559	0.551	0.917
	Dominant	0.83(0.81–0.86)	<0.001	1.5%	0.513	0.340	0.466
	Recessive	0.79(0.75–0.84)	<0.001	0.9%	0.509	0.377	0.029
	Additive	0.86(0.84–0.88)	<0.001	2.6%	0.517	0.238	0.251
Small size							
N = 12	T vs C	0.86(0.81–0.90)	<0.001	0%	0.811	0.271	0.373
	TT vs CC	0.73(0.65–0.82)	<0.001	0%	0.502	0.502	0.732
	CT vs CC	0.86(0.80–0.92)	<0.001	4.6%	0.400	0.060	0.150
	Dominant	0.83(0.78–0.89)	<0.001	0%	0.669	0.078	0.115
	Recessive	0.79(0.70–0.88)	<0.001	15.6%	0.291	0.380	0.631
	Additive	0.85(0.81–0.90)	<0.001	0%	0.787	0.405	0.537
NSCLC							
	T vs C	0.86(0.81–0.90)	<0.001	11.9%	0.338	0.480	1.000
	TT vs CC	0.71(0.63–0.80)	<0.001	0%	0.472	0.842	0.806
	CT vs CC	0.88(0.82–0.95)	<0.001	45.7%	0.188	0.367	0.462
	Dominant	0.85(0.79–0.91)	<0.001	35.8%	0.183	0.382	0.462
	Recessive	0.75(0.67–0.84)	<0.001	0%	0.462	0.872	1.000
	Additive	0.86(0.82–0.91)	<0.001	0%	0.466	0.654	0.806

To provide a more precise estimate of the effect of rs402710 on NSCLC, we combined our current study and previously published studies which displayed detailed information about NSCLC, including 5 studies of 6060 NSCLC cases and 8678 controls. The heterogeneity in heterozygous (*I^2^* = 45.7%) and dominant (*I^2^* = 35.8%) model among NSCLC studies was moderate, which might be due to different study design and genotyping methods. Under fixed-effects model, the significant association with decreased lung cancer risk was exhibited in all genetic models.

#### Sensitivity analyses and Cumulative meta-analyses

**Figure 2:**
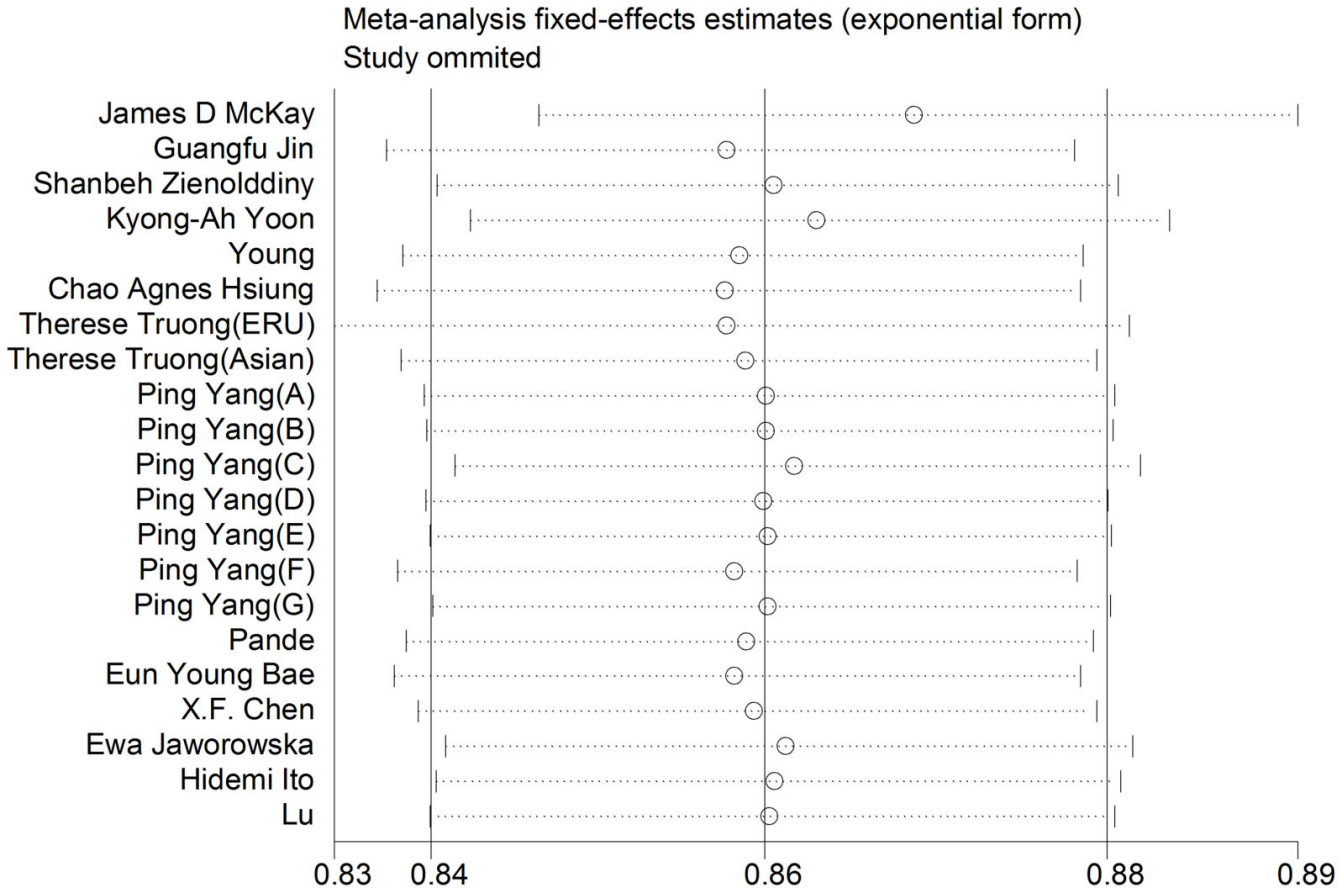
Sensitivity analysis of allelic model.

To assess the effect of individual study on the pooled estimate, we performed a sensitivity analysis by omitting each study in turn. As shown in Table 6, the result of the allelic model was particularly robust when we eliminated each study. There was almost no change of the ORs and 95%CIs after each deletion (Figure 2). Analogous results existed in other genetic models and no single study conspicuously changed the pooled ORs.

**Table 6:** Sensitivity analysis of the allelic model.

Study omitted	OR(95%CI)	*P*	*I^2^*	*P* for Heterogeneity
James D McKay(2008)	0.87(0.84–0.89)	<0.001	0%	0.920
Guangfu Jin(2009)	0.86(0.84–0.88)	<0.001	0%	0.842
Shanbeh Zienolddiny(2009)	0.86(0.84–0.88)	<0.001	0%	0.780
Kyon-Ah Yoon(2010)	0.86(0.84–0.88)	<0.001	0%	0.850
Young(2010)	0.86(0.84–0.88)	<0.001	0%	0.854
Chao Agnes Hsiung(2010)	0.85(0.83–0.88)	<0.001	0%	0.806
Therese Truong(ERU)(2010)	0.86(0.83–0.88)	<0.001	0%	0.777
Therese Truong(Asian)(2010)	0.86(0.84–0.88)	<0.001	0%	0.783
Ping Yang(A)(2010)	0.86(0.84–0.88)	<0.001	0%	0.770
Ping Yang(B)(2010)	0.86(0.84–0.88)	<0.001	0%	0.770
Ping Yang(C)(2010)	0.86(0.84–0.88)	<0.001	0%	0.842
Ping Yang(D)(2010)	0.86(0.84–0.88)	<0.001	0%	0.770
Ping Yang(E)(2010)	0.86(0.84–0.88)	<0.001	0%	0.770
Ping Yang(F)(2010)	0.86(0.84–0.88)	<0.001	0%	0.893
Ping Yang(G)(2010)	0.86(0.84–0.88)	<0.001	0%	0.773
Pande(2011)	0.86(0.84–0.88)	<0.001	0%	0.791
Eun Young Bae(2011)	0.86(0.84–0.88)	<0.001	0%	0.826
XF Chen(2011)	0.86(0.84–0.88)	<0.001	0%	0.813
Ewa Jaworowska(2011)	0.86(0.84–0.88)	<0.001	0%	0.798
Hidemi Ito(2012)	0.86(0.84–0.88)	<0.001	0%	0.778
Lu(2012)	0.86(0.84–0.88)	<0.001	0%	0.771
Combined	0.86(0.84–0.88)	<0.001	0%	0.818

**Figure 3:**
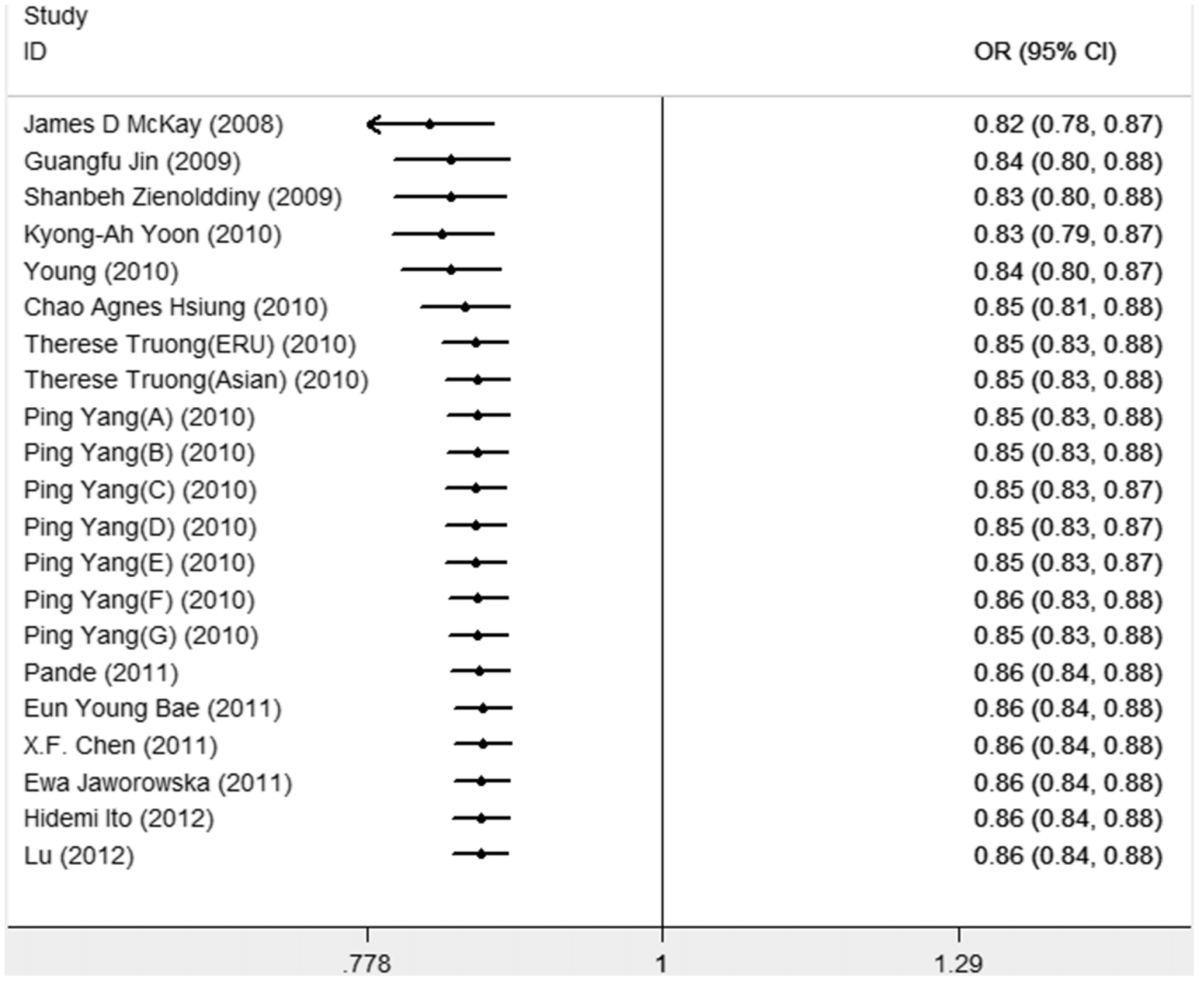
Cumulative meta-analysis of association rs402710 with lung cancer under allelic model.

Cumulative meta-analyses were also conducted in all genetic models via assortment of studies by chronological order. As shown in Figure 3, in the allelic model, the 95% CIs for the pooled OR became gradually narrower with each accumulation of more studies, which indicated that the precision of the estimation was progressively boosted by continual adding more sample. Similar results were also observed in other genetic models.

#### Publication bias

As reflected by the funnel plot, the Egger's test and the Begg's test, there was no publication bias under all genetic models (*P _Egger_*>0.05, *P _Begg_*>0.05) and funnel plot was visually symmetrical.

#### The bioinformatics analyses of rs402710

Only one of the three bioinformatics tools, “F-SNP” forecasted that the SNP was likely to participate in transcriptional regulation with a low score.

## Discussion

In our case-control study, a significant association between the SNP rs402710 and lung cancer risk was revealed under heterozygous, dominant and additive model in Chinese population. Additionally, the following meta-analysis integrating our current study and 13 previous studies with a total of 31811 cases and 36333 controls, concordantly demonstrated the association between rs402710 and lung cancer under all six genetic models. Subsequently, with each accumulating of more chronological data, cumulative meta-analysis displayed the more precise effect of this variant with 95% CIs of the pooled ORs becoming progressively narrower. Before and after the deletion of each study, estimates of all genetic models were similar in sensitivity analysis, manifesting the robust stability of the meta-analysis. Little publication bias was detected.

The rs402710 is located in intron 4 of the *CLPTM1L* gene on chromosome 5p15.33, which contains two biologically relevant genes for lung cancer, TERT (telomerase reverse transcriptase) and *CLPTM1L*. The copy number gain of this region of chromosome 5p is the most frequent cytogenetic event in the early stages of NSCLC [41]. However, for a long time, the functions of *CLPTM1L* gene were poorly understood, and it was observed as a cisplatin-resistance factor in ovarian cancer-cell lines which can't be taken as a function of *CLPTM1L* gene on oncogenesis [42]. Remarkably, published in June of 2012, James MA et al [43] demonstrated that *CLPTM1L*, as an overexpressed protein in lung tumor cells, protected from genotoxic stress induced apoptosis through regulation of Bcl-xL, which implicated that the anti-apoptotic *CLPTM1L* function may be a potential mechanism of susceptibility to lung tumorigenesis.

The *CLPTM1L* gene variant rs402710 is associated with higher DNA adduct formation in tumor adjacent lung tissue [38]. Levels of these DNA adducts (formed by polycyclic aromatic hydrocarbons and aldehydes found in cigarette smoke) can be used as a biomarker accounting exposure to tobacco carcinogen. It is possible that the rs402710 variant may enhance formation and persistence of DNA adducts. However, the association between this variant and the *CLPTM1L* gene is not clear. Through bioinformatics analysis, we found that this variant may participate in transcriptional regulation. But the effect of the SNP in lung tumorigenesis needs to be affirmed by further studies.

The susceptibility locus rs402710 was firstly identified in a GWA study of Caucasian population by Mckay JD et al [12], however, the follow-up replication studies showed inconsonant results. The association between rs402710 and lung cancer has been respectively confirmed in some of the genetic models in Japan population [18], Korea population [19], [40] and mixed Asian population [17], but two replication studies in Chinese population failed to carry out the similar results [20], [21]. Although a meta-analysis about this SNP rs402710 by Simone Mocellin et al [44] drew a positive conclusion, it contained 10 studies before 2011 and only demonstrated allelic model. In our case-control study in Chinese population, the association between rs402710 and protective effect of lung cancer was identified in heterozygote, dominant and additive models but failed in allelic, homozygous and recessive models, may be due to the small sample size of the study. In NSCLC, it is remarkable that larger effect of the minor allele T was detected in NSCLC than that in overall lung cancers. Additionally, the following meta-analysis comprising 31811 cases and 36333 controls consistently suggested the significant association of rs402710 with protective effect of lung cancer in all genetic models. After stratification by ethnicities or study sample size, the significant associations between SNP rs402710 and lung cancer were identified in all genetic models of all subgroups. However, the genetic effect on Caucasian was stronger than that of Asian under every genetic model, likely relating to different allele frequencies between Asian and Caucasian. In NSCLC, the significant associations with decreased lung cancer risk were explored in all genetic models, which further supported our findings in case-control study.

Although no heterogeneity was detected in all genetic models among included studies, stratified analyses was still performed to explore the differences between ethnicities or study sample size. In Caucasians, no significant heterogeneity was detected, whereas the heterogeneity in heterozygous and recessive model among Asian studies increased from no heterogeneity grade to moderate grade. The same phenomenon, that the heterogeneity between large sample size studies removed and the small group increased, existed after stratified by study sample size, which may be due to the sampling error in small studies. Furthermore, the sensitivity analysis and publication bias estimation illustrated the current results of this meta-analysis were robust.

Despite the clear strength of the current study possessing enough power, some limitations should be seriously considered. Firstly, the sample size of our case-control study was relatively small. Secondly, absent data or insufficient data restricted us to perform further analysis. Thus we couldn't analysis the association between the SNP rs402710 and lung cancer subtypes including adenocarcinoma, non-small cell lung cancer, small cell lung cancer and others. Finally, lung cancer is a complex disease caused by both genetic and environmental factors, but the gene-environment interaction can't be evaluated owing to the absence of environmental information.

In conclusion, the current case-control study and the follow-up meta-analysis helped to strongly clarify the significant association between rs402710 and lung cancer, and our study implied that the variant may yield stronger effect on NSCLC and never smokers. However, it is needed to implement fine-mapping of 5p15.33 region or function analysis to identify causal variant.

## Supporting Information

Figure S1 Follow chart of study selection.
(TIF)

Checklist S1 PRISMA checklist.
(DOC)
