# Supplementary figures and images for: The SNP rs402710 in 5p15.33 Is Associated with Lung Cancer Risk: A Replication Study in Chinese Population and a Meta-Analysis
Source: PLoS One. 2013 Oct 23;8(10):e76252. doi: 10.1371/journal.pone.0076252 (PMC3806805; doi:10.1371/journal.pone.0076252)

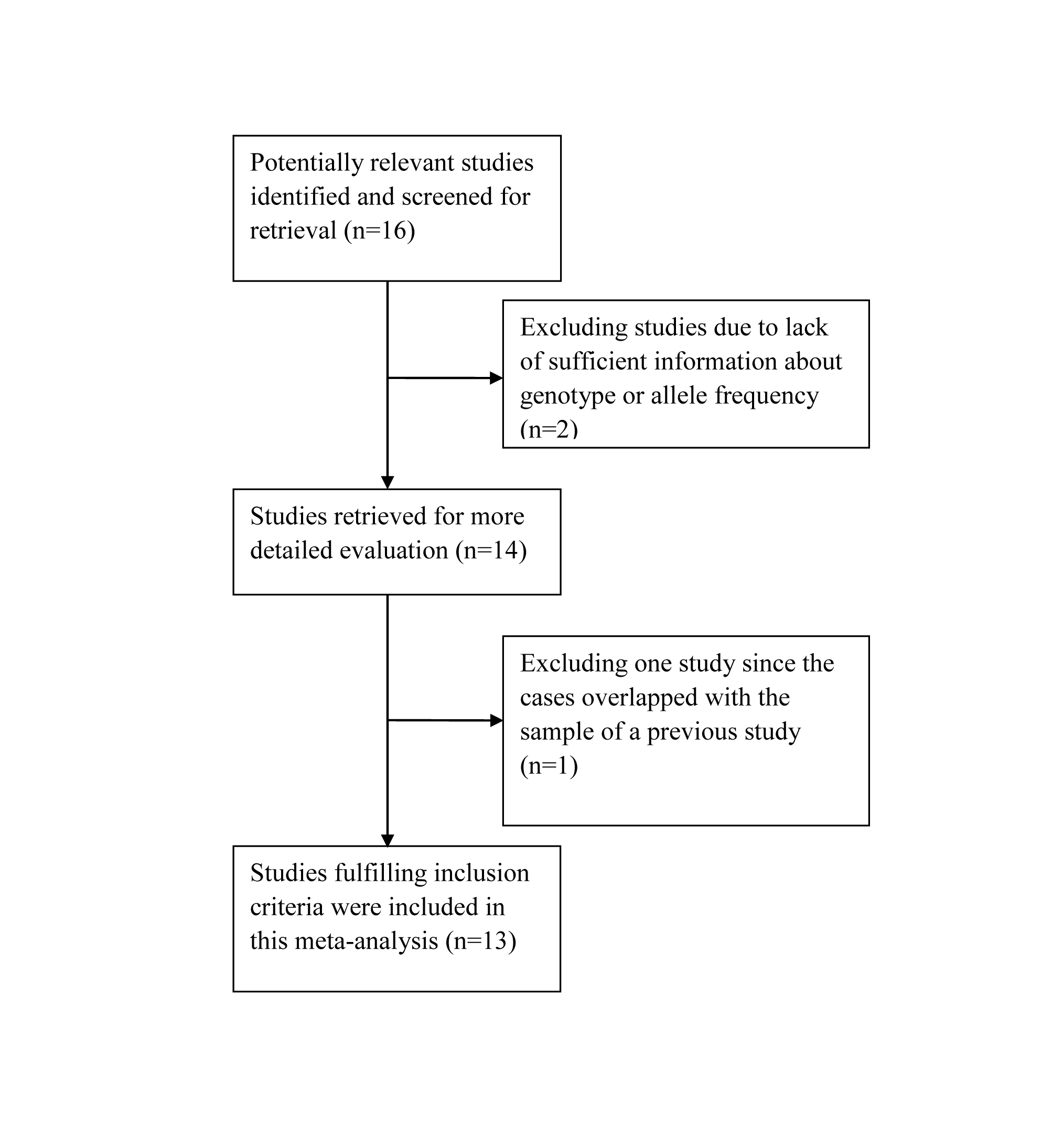

Supplement: Figure S1 — Follow chart of study selection. (TIF) [file pone.0076252.s001.tif]
